# Magma pressure discharge induces very long period seismicity

**DOI:** 10.1038/s41598-021-99513-4

**Published:** 2021-10-08

**Authors:** M. Ripepe, D. Delle Donne, D. Legrand, S. Valade, G. Lacanna

**Affiliations:** 1 Department of Earth Science, University of Florence, Firenze, Italy; 2 Istituto Nazionale Geofisica e Vulcanologia, Osservatorio Vesuviano, Napoli, Italy; 3 Department of Volcanology, Universitad Nacional Autonoma de Mexico, Mexico City, Mexico

**Keywords:** Volcanology, Geophysics

## Abstract

Volcano seismicity is one of the key parameters to understand magma dynamics of erupting volcanoes. However, the physical process at the origin of the resulting complex and broadband seismic signals remains unclear. Syn-eruptive very long period (VLP) seismic signals have been explained in terms of the sudden expansion of gas pockets rising in the liquid melt. Their origin is linked to a magma dynamics which triggers the explosive process occurring before the explosive onset. We provide evidence based on acoustic, thermal, and ground deformation data to demonstrate that VLP signals at Stromboli are generated at the top of the magma column mainly after the explosion onset. We show that VLP amplitude and duration scale with the eruptive flux which induces a decompression of 10^3^–10^4^ Pa involving the uppermost ~ 250 m of the feeding conduit. The seismic VLP source represents the final stage of a ~ 200 s long charge and discharge mechanism the magma column has to release excess gas accumulated at the base of a denser and degassed magma mush. The position of the VLP seismic source coincides with the centroid of the shallow mush plug and tracks elevation changes of the magma free surface.

## Introduction

Open vent volcanoes are generally characterized by a sustained and restless degassing dynamics which generates a large variety of seismic signals. Moderate explosive activity is generally explained in terms of nucleation, ascent, and bursting of a large gas slug^1^, which is thought to be responsible for the origin of the seismic VLP activity on many volcanoes on the globe^2–11^. However, the genesis of VLP seismic signals is still matter of debate.

Seismic VLP signals are commonly recorded on a large variety of magmatic systems, ranging from high-viscosity andesitic^9,12^ to low-viscosity basaltic volcanoes^6,13,14^. However, VLP signals share similar seismic characteristics^2–11,15,16^. Their amplitude quickly decays moving away from the craters and are thus mainly recorded at a distance of few kilometers from the vent. Frequency range is confined in the 0.01–0.2 Hz band and have wavelength tens of kilometers long. Location of the VLP source is generally shallow and confined within the first hundreds of meters below the craters. Waveform of seismic VLP signal is generally simple and with a strong rectilinear polarization vector pointing towards the craters, typical of P-waves. These similarities suggest a link to a more general dynamics which seems to be common to many active volcanoes in the world^3–5,13,15,16^.

When seismic VLPs are observed as associated with explosive activity and/or gas puffing at the surface their origin is commonly explained as the opening-closing of a crack induced by the sudden volume expansion within the fluid melt several seconds (between 3 and 60 s) before the explosion^2–11,13,15–17^. However, seismic VLPs are not always associated with visible explosive activity^18,19^, thus suggesting different mechanisms acting inside the conduit^5^. Here we use data collected at Stromboli volcano (Aeolian Islands, Italy) to shed light on the origin of syn-eruptive VLP seismic activity using a multiparametric approach.

Explosive activity at Stromboli volcano is generally moderate (VEI = 1) and persistent through time^20^ repeating at a mean rate of ~ 13 events/h^21^ (termed ‘normal activity’^20,21^) which provides evidence of an efficient degassing mechanism of the magma column, typical of most basaltic open-conduit volcanoes. At Stromboli, seismic VLP waveforms repeat in time in well-constrained families^2,18^ indicating a source mechanism typical of a non-destructive process acting in a quasi-stationary dynamics^2,18,22,23^. Semblance^18,24^ and seismic moment tensor inversion^2^ locate the centroid of the VLP seismic source at a shallow ~ 220–260 m depth below the craters and at ~ 160 m NW off the vents^2,18^ within the Sciara del Fuoco. This stability in time suggests the presence of a persistent physical constraint on the source process and location of the VLPs. At this depth, a geometrical change in the feeding conduits is believed to induce the sudden expansion of the rising gas slug and the consequent magma decompression recorded by the seismometers as VLP signal^2,25^. Moment tensor inversion and VLP source location are thus interpreted as evidence of an inclined dyke which is responsible for the sudden expansion of the slug while rising to the surface^2^. This interpretation has contributed to picturing the geometry of the shallow feeding system at Stromboli and to explaining the VLP source process as a pressure source within an opening/closing crack embedded in the volcanic edifice^2,4,26^. This model times the source of the VLP to seconds before the explosion (bottom-up model^2^) and would require a very fast ascent gas velocity (50–100 m/s)^10,27^ to explain the link with the explosive process as evidenced by camera and infrasound records of strombolian eruption at Stromboli, Kilauea^28^, Fuego^4^, Aso^10^, and Erebus^3^ volcanoes.

At the scale of several years, at Stromboli, the position of the VLP seismic source, as monitored by the dip of the VLP polarization seismic vector, shows large (~ 10 degrees) relative changes^18,29–31^ that are well correlated with long-term changes in the topographic elevation of summit craters^30^. Larger fluctuations in the VLP polarization vector occur during lateral effusive eruptions when strombolian activity is no longer visible at the summit craters. Deepening of the polarization vector has been correlated to the discharge of the magma shallow reservoir and explained by the lowering of the magma column within the feeding conduits^30–32^.

In this paper we present evidence incorporating thermal imagery, acoustic pressure, and ground deformation data to produce a new physical model that explains the seismic VLP as the final stage of recurrent charge/discharge of the excess gas accumulated in the shallow part of the feeding conduit, connecting the VLP source to the surface of the magma column and to the eruptive flux.

## Results

### The seismic VLP wavefield

**Figure 1:**
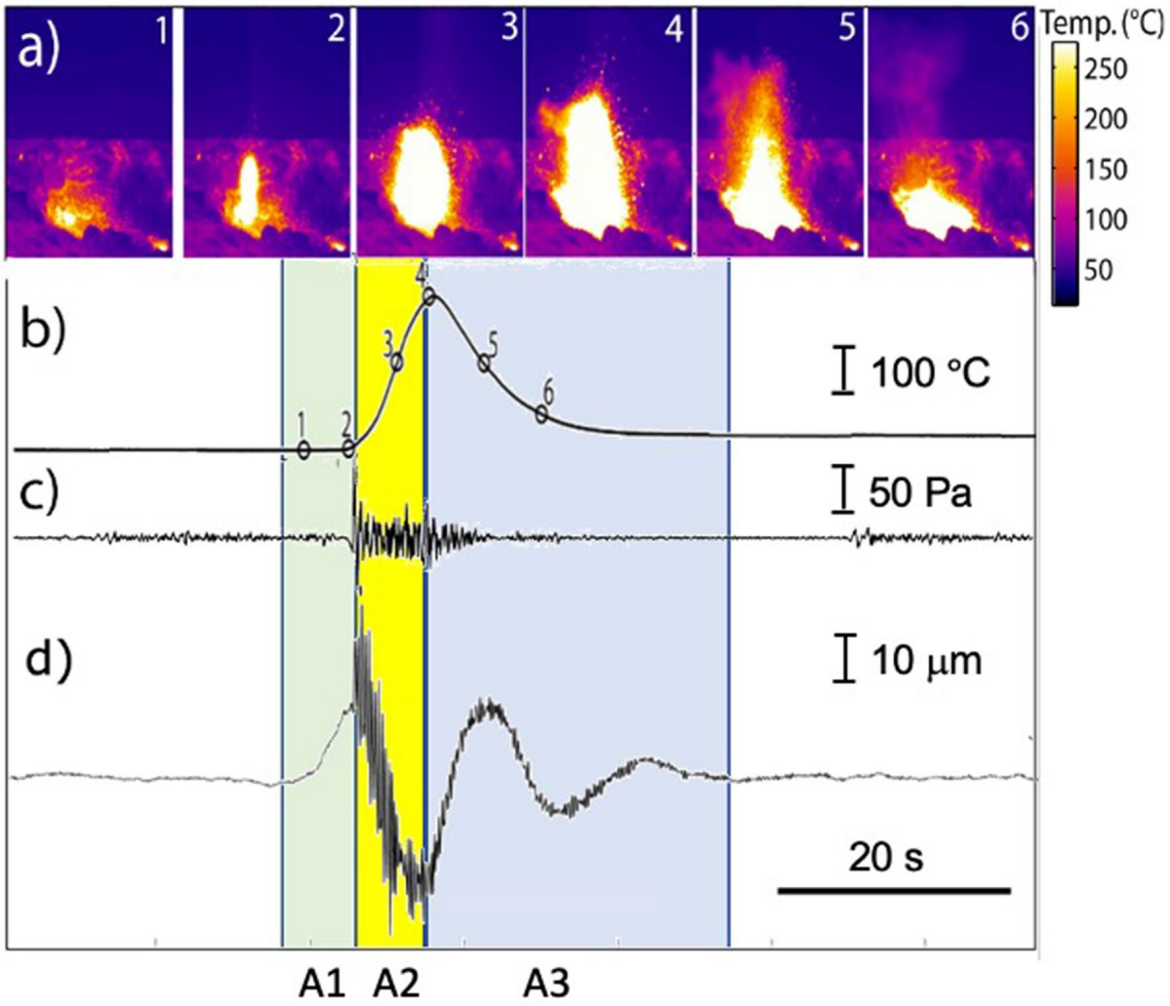
(**a**) Snapshots of thermal frames capturing a normal strombolian explosion and its associated (**b**) thermal signal, here displayed as the average temperature within the camera’s field of view, (**c**) infrasonic signal and (**d**) vertical component of the seismic VLP ground displacement recorded at the S11 station (Figure S2). Numbers indicate at which position of the thermal signal every frame is associated with. Infrasound has been corrected for the propagation time relative to the source-station distance. Explosive onset is marked by the increase of thermal emission (from #2 to #4) and coincides with the infrasonic onset and the beginning of the deflation phase of VLP displacement. Colored areas mark: (A1) the pre-explosive phase characterized by the compressive seismic VLP onset; (A2) main VLP dilatation phase concomitant with the strombolian explosion visible at the surface as detected by the thermal camera, and (A3) the post-explosive oscillation of the VLP coda. It is worth noting that seismic VLP signal also shows a high-frequency signal at the beginning of the explosive onset attributable to explosion-generated and scattered high-frequency body and/or surface waves.

We here define VLP seismic displacement as the integration of ground velocity filtered between 0.05 and 0.2 Hz. In harmony with previous experiments^2,18^ we derive two different families of VLP seismic signals (Figure S1). The vertical seismic component of both family shows coherent polarity for all stations and is characterized by an initial low amplitude and compressional onset (A1 in Fig. 1) lasting ~ 5–6 s that is followed by a second, larger amplitude, dilatation phase (A2 in Fig. 1) lasting ~ 4–12 s (see also Ref.^2,18,24^). This energetic second part of the waveform is sometimes followed by a coda 20–30 s long (A3) characterized by one to two low amplitude oscillations.

Once the VLP wavefield is rotated into the vent-radial direction, ground displacement polarities at the stations are coherent and are consistent with a roughly isotropic source producing an alternating compression-dilatation mechanism that is nearly symmetrical with respect to the crater position^2,24^. Although Stromboli volcano is characterized by three main active vents (Supplementary note S1) well-separated in space (~ 200 m), the VLP radiation patterns do not seem to depend on the particular exploding vent, but are quite stable^18^ and oriented radially with respect to the central crater area (Figure S1).

### VLP seismicity and thermal flux

Thermal radiance reflects two different phases of the explosive dynamics. While the initial increase in temperature is due to hot ash and scoria fragments ejected during the gas thrust phase (A2, Fig. 1), the waning phase of the thermal temperatures (A3 Fig. 1) is linked to plume expansion during the buoyancy of the gas-fragments cloud^33–35^. Explosive eruptions are detected as variation of thermal radiance providing estimates of the onset, duration, and thermal mass flux associated with the explosion. In harmony with previous observations made at Stromboli^2,18^, as well as on other volcanoes^3,11,28^, thermal radiance shows that seismic VLP signals are well correlated with explosive events, as they always start at the end of VLP seismic compression phase (A1 in Fig. 1a).

**Figure 2:**
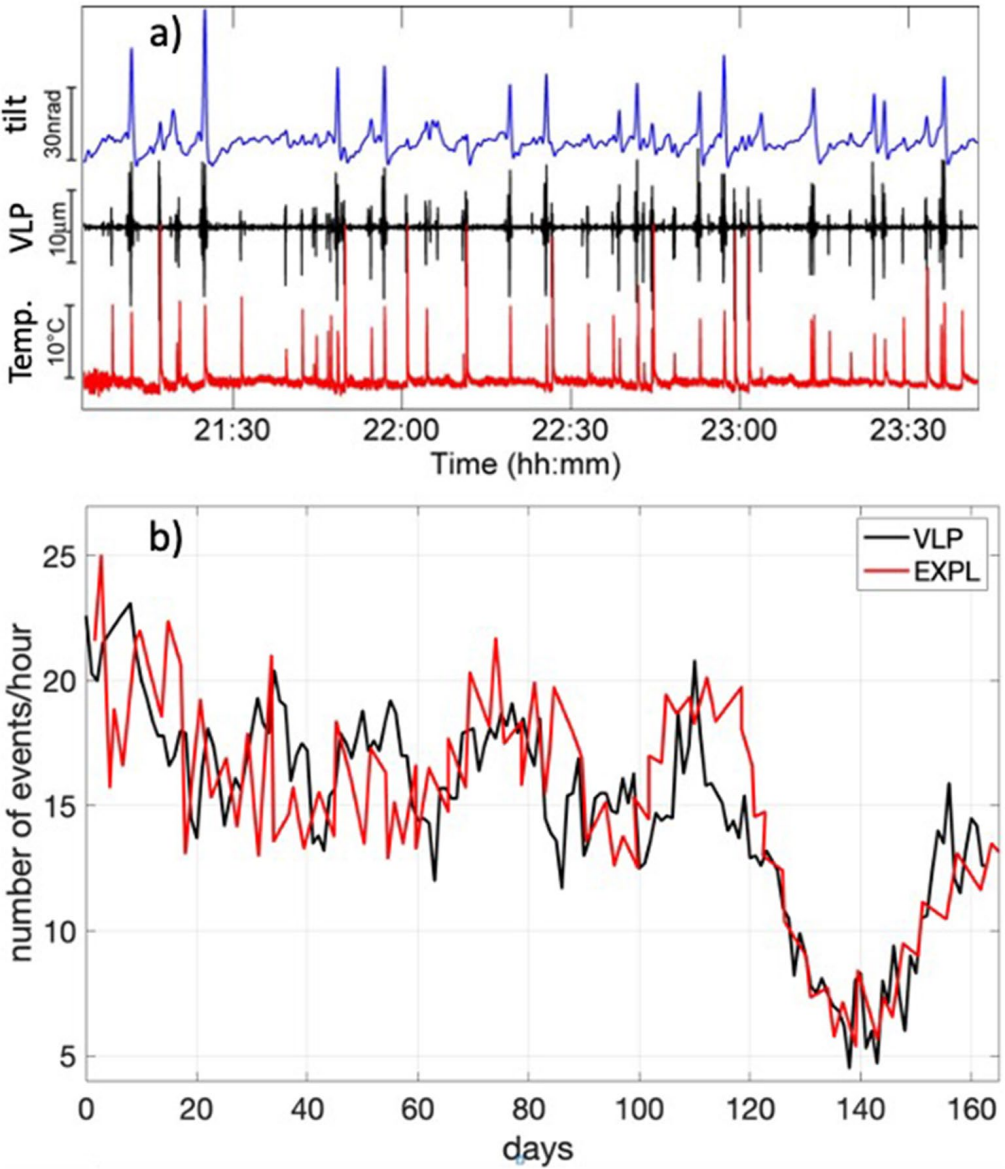
(**a**) Two and a half hour-long record of the ground tilt radial component (blue line), seismic VLP vertical displacement (black line) and thermal emissions (red line) revealing the strong link among VLP seismicity and the explosive process. Explosions detected as thermal transients are always associated with a seismic VLP signal which sits on top of the repetitive ~ 100–200 s long ground inflation-deflation cycles. (**b**) Long term correlation between the daily average of number of VLP events per hour (black line) and the number of explosions per hour detected by the permanent thermal camera (red line) at ROC site (Figure S2). The extremely coherent fluctuations over days-to-month time scale between these two parameters demonstrate the fundamental association of VLP seismicity with strombolian explosions.

The link between VLP seismicity and explosive activity is better evidenced in the long-term time scale when the daily numbers of VLP^30,31,35^ are compared to the number of daily explosions observed by the thermal camera (ROC in Figure S2) of the permanent network (Fig. 2b). Thermal transients and VLP rates change together over days to months and highlight the fluctuations of the explosive level during normal activity (Fig. 2b).

**Figure 3:**
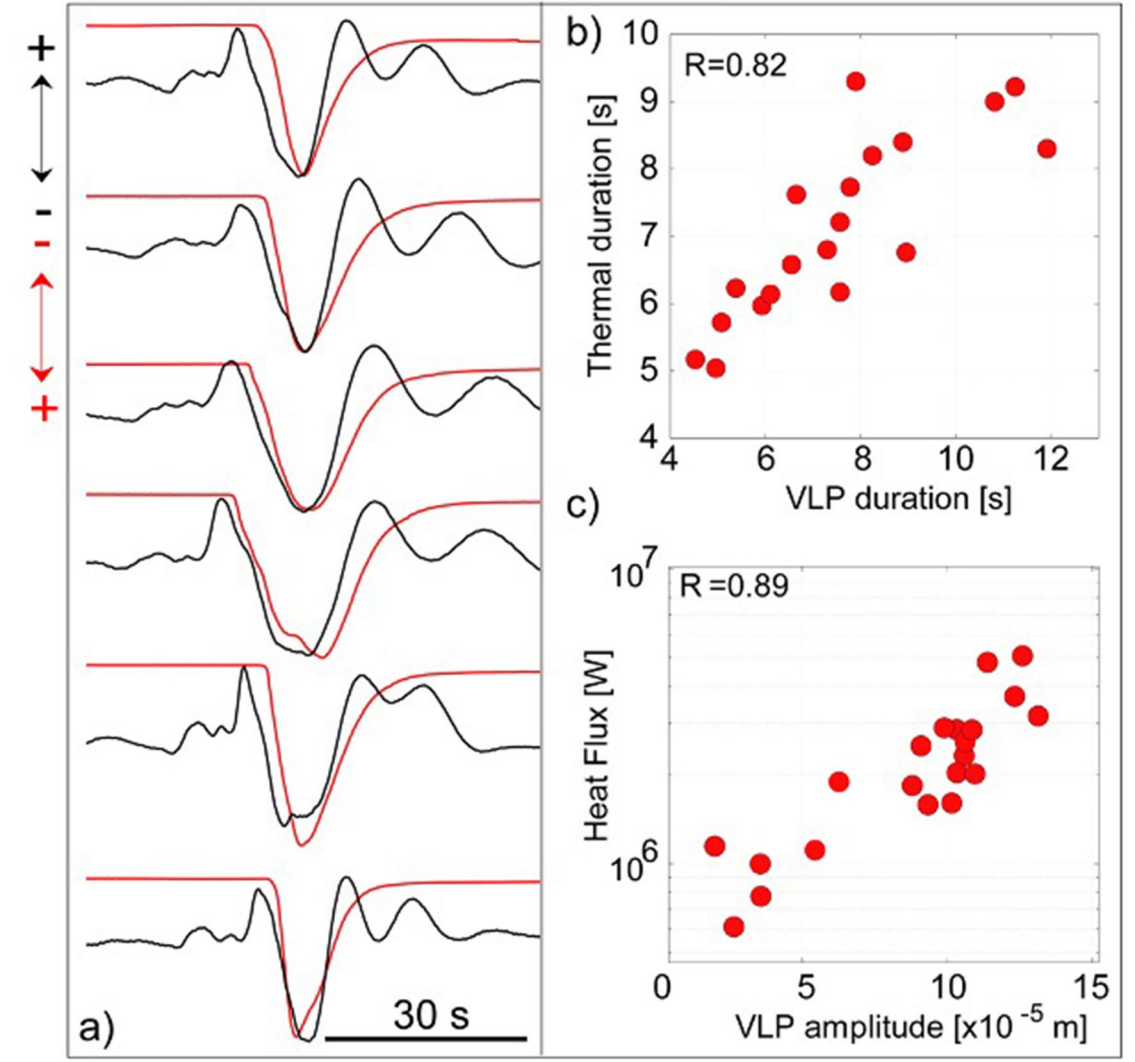
(**a**) VLP (black line) and thermal waveforms (red lines) relative to six strombolian explosions at both NE and SW craters (+ and − arrows in upper left corner of the figure indicate signal polarity; note that we inverted the thermal signal to demonstrate its correlation with the VLP signal). The VLP dilatation phase (A2 in Fig. 1) starts at the onset of thermal signal and has the same duration as the gas-thrust driven jet phase duration of the explosion. (**b**) Duration and (**c**) amplitude of the VLP (A2) dilatation phase, for 19 explosions, show a high correlation (R = 0.82 and 0.89, respectively) with thermal duration and heat flux.

Using high-frame rate (50 Hz) thermal images^35^ taken in line of sight with the exploding vent (PSF, Figure S2), we found that the onset of the thermal signal marks the transition between the compressional (A1) and the large dilatation (A2) phase of the seismic VLP (Fig. 1). In particular, the duration Δ*t* of the thermal gas-thrust phase (from the onset, #2, to the maximum recorded temperature, #4, in Fig. 1) shows (Fig. 3) a very good correlation of R = 0.82 (Pearson correlation coefficient) with the duration of the A2 VLP dilatation phase measured on the vertical component of the ground displacement (Fig. 3b). The correlation becomes straightforward (R = 0.89) when the heat flux *Φ*, and the VLP amplitude of the A2 phase are compared (Fig. 3c).

Here we have considered the maximum of the heat fluxes *Φ* (in W) associated with explosions which have been calculated as, $\Phi ={A}_{FOV}\sigma \epsilon \left({T}_{E}^{4}-{T}_{BKG}^{4}\right)$ where *T*_*E*_ is the peak of the temperature integrated within of the camera’s field of view (*A*_*FOV*_), *T*_*BKG*_ is background temperature, *σ* is the Stefan-Boltzmann constant, and *ε* is emissivity (~ 0.96^37^).

The thermal camera therefore indicates that explosive dynamics tracks the amplitude and duration of the A2 dilatation phase (Figs. 1 and 3a) of the seismic VLP waveform. Such temporal correlation between thermal imagery data and VLP waveform is observed nearly for all explosions and it constitutes a key evidence for linking the source process to explosive activity. These observations suggest that the violent gas expansion during the gas thrust phase (Fig. 1), produces a downward momentum transferred to the volcanic edifice that is detected by seismometers as the VLP displacement field^38,39^ (Fig. 1d).

### VLP seismicity and acoustic emission

Acoustic waves provide a second body of evidence to associate the seismic VLP signal with explosive process occurring at the surface. Acoustic pressure (*p*) is explained in terms of the rate of acoustic volumetric flux (*Q*_*i*_)^40–42^ generated during the violent expansion of the gas-fragments mixture within the conduit, first, and into the atmosphere afterwards. Acoustic pressure thus reflects the history by which the magmatic column is reducing its internal pressure, and is proportional to the rate at which gas expands outside the conduit^35,43^. Here, we show that when the acoustic signal is corrected for the propagation time between the source and the station, the onset of the acoustic wave coincides with the beginning of the seismic VLP large (A2) dilatation phase (Fig. 1). Considering the topography, the conduit geometry and the scattering effects on the infrasonic amplitude *p*(*t*), the volumetric flux inside the conduit *Q*_*i*_ can be calculated as^43^:

1 $${Q}_{i\left(t\right)}=\frac{2\pi r}{\left(1+\left|{R}_{a}\right|\right)\rho \alpha 1{0}^{\left(\frac{IL}{20}\right)}}{\int }_{0}^{t}p\left(t+\frac{r}{c}\right)dt$$

where *ρ* = 1.1 kg/m^44^ is the air density, *r* (~ 300 m) is the crater-station slant distance, *c* = 340 m/s is the sound speed in air, *IL* is the insertion loss of ~ 1 dB estimated for the specific source-station path, and |*R*_a_| (= 0.96) is a parameter which takes into account the vent exit radius and frequency content of infrasonic signal. We here considered an isotropic radiation pattern (*α* = 1), which has proven to be valid for explosions at Stromboli^43^.

**Figure 4:**
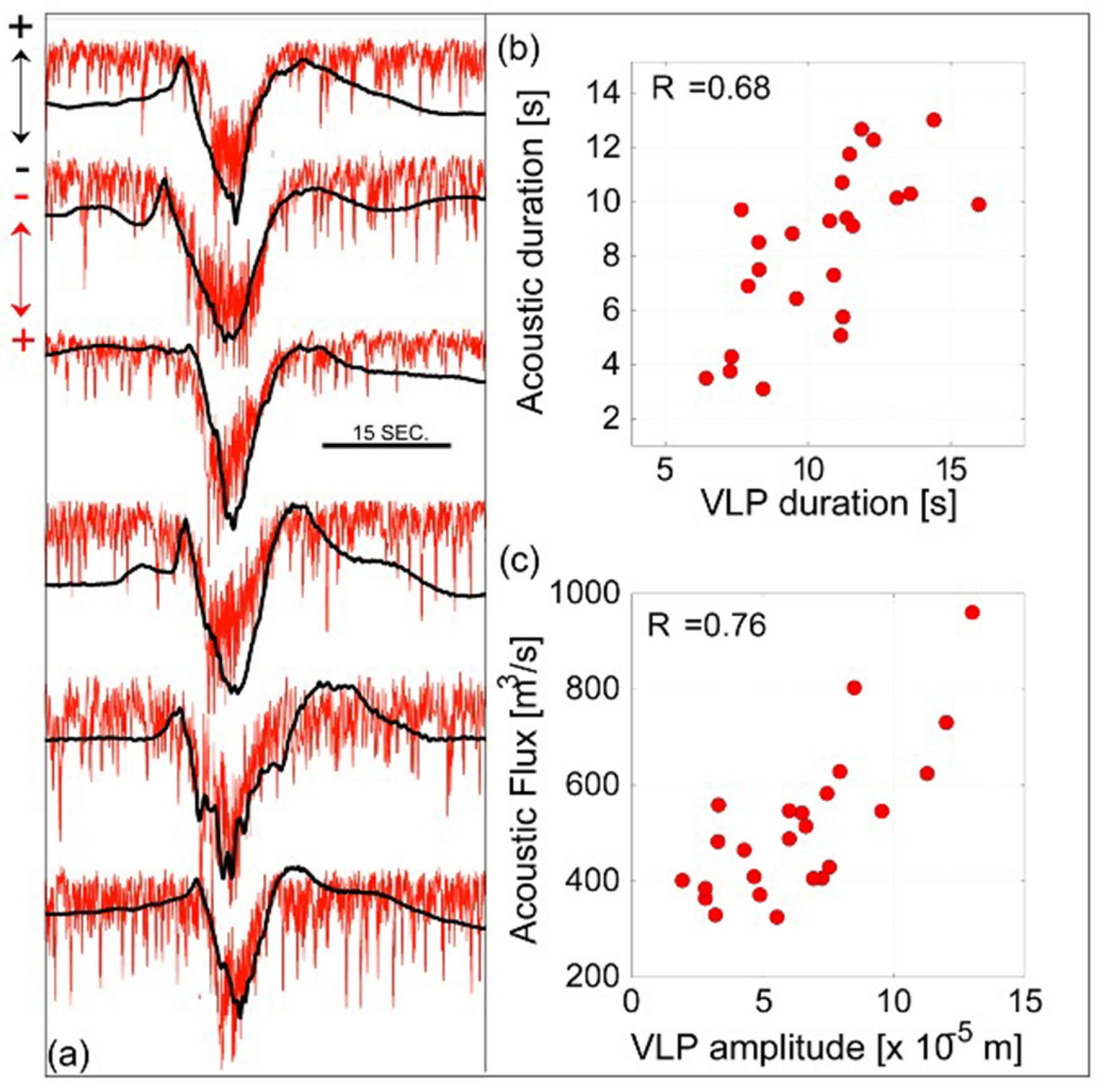
(**a**) Seismic VLP signal (black line) shows a strong correlation with volumetric fluxes (red line) derived from the acoustic signal (+ and − arrows in upper left corner of the figure indicate signal polarity). (**b**) Duration of the acoustic flux of 24 explosions is well correlated (R = 0.68) to duration of the seismic VLP (A2) phase. Volumetric fluxes derived from the acoustic signals (Eq. 1) show a positive correlation (R = 0.76) with the amplitude of the VLP dilatation phase A2.

Our results (Fig. 4) indicate that acoustic volumetric flux ranges between 200 and 1500 m^3^/s and is fully comparable with gas flux directly measured by UV camera^45,46^. Acoustic flux correlates (R = 0.76) with the amplitude of the VLP seismic A2 dilatation phase and its duration scales with the total duration of the acoustic signal (R = 0.68). Consistent with the thermal analysis, these observations support the hypothesis that the VLP phase A2 (Fig. 1) originates during the gas thrust regime of the explosive dynamics, and its amplitude reflects the upward volumetric flux of the explosive fluid.

### VLP seismicity and ground deformation

Seismic VLP signals are at Stromboli always associated with a well resolved deformation of the ground (Fig. 2a). This process repeats in time for every explosion (Fig. 2a) in a cyclic charge/discharge process^47^. Considering a propagation P-wave velocity of ~ 3500 m/s^2^, the wavelength of the VLP seismic radiation is tens of km (~ 40 km), which compared with the distance from the source (< 500 m), indicates that we are in a near-field condition. In such a case, seismic VLP displacement reflects the source time function and can be considered to be a time dependent quasi-static volume displacement of the source^2,3,16,48–50^. Near-field conditions also make it possible to convert the low frequency band seismic signal into ground tilt using analytical solutions^47,51,52^.

**Figure 5:**
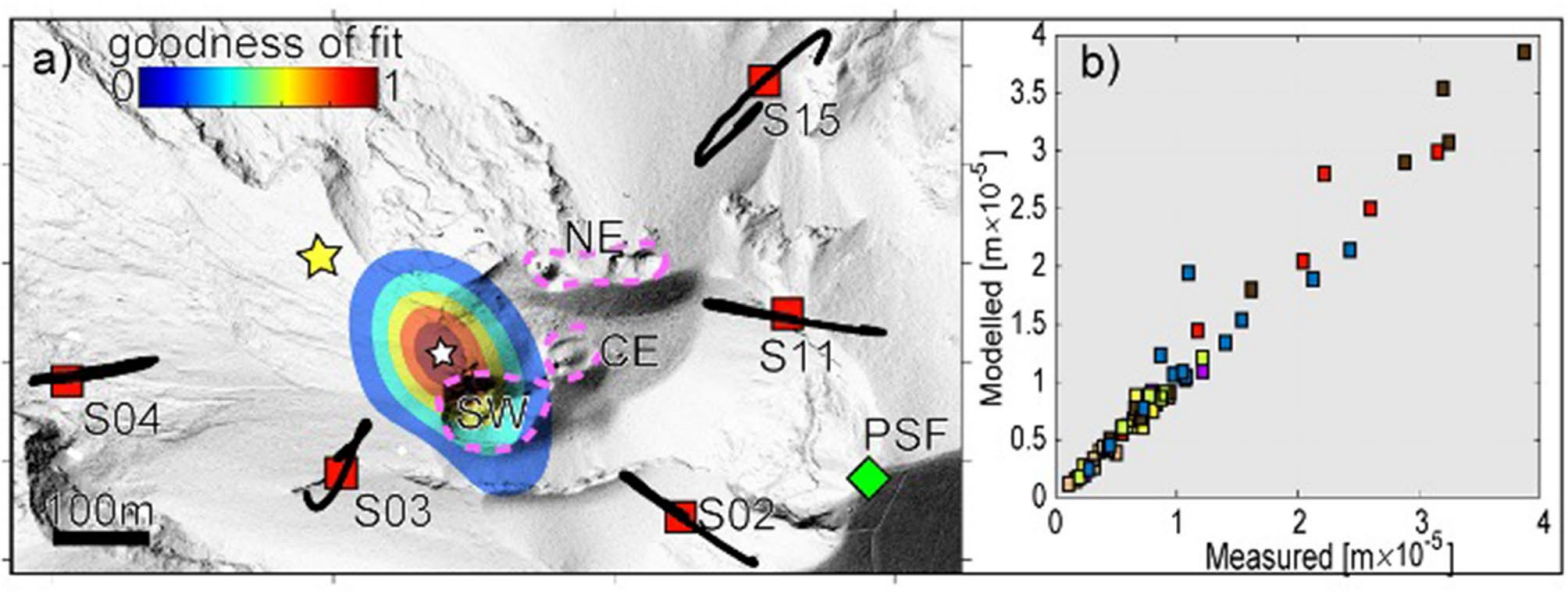
(**a**) Average source location and misfit map for 40 VLPs calculated by the grid search method using the open-conduit deformation model. Source location of VLP using the quasi-static displacement (white star) is compared with the location of the VLP derived by seismic moment inversion (yellow star)^2^. Source location is stable for all the considered VLPs and is found to extend 250 m below the surface close to the SW crater (Figure S1e, f). Average seismic horizontal VLP particle motions calculated for VLPs recorded in one day (black lines) are consistent with the position of the source (white star). Stations used for the source location are shown as red squares, while PSF (green diamond) indicates the position of the thermal image acquisition used for Figs. 1 and 3; (**b**) Forward test applied on these 40 VLP events considering an open-conduit-shaped source yields a correlation coefficient R > 0.95 between measured and modeled vertical VLP displacement. The map in **a**) was created using Matlab ver. 7.5.0.338 (R2007b).

We argue that the VLP A2 dilatation phase is reflecting the vertical force induced by the mass flux associated with the explosive ejection of the gas-fragments mixture, and we thus apply a model of quasi-static deformation to estimate the location and the associated volumetric change. We used data recorded by a network of five temporary seismic stations located within 300 m from the active vents with a ~ 70% azimuthal coverage around the craters (Fig. 5). To the best of our knowledge, these are the first measurements of the VLP wavefield at Stromboli made inside the crater area using a broadband network. This allowed us to record VLP signals in the very near-field with the best azimuthal coverage as possible, and to constrain the source position by minimizing as much as possible the effect induced by the network geometry.

Considering the open-conduit model^53^, the vertical VLP displacement *u*_*z*_ can be defined as proportional to the volumetric change *ΔV* of an *L*-extended conduit:

2 $${u}_{z}=\frac{5}{3}G\frac{\Delta V}{\pi L}$$

where *G* is the Green’s function^54^, which depends on the position of the conduit relative to the stations. We apply a grid search procedure on a 3D space within the seismic network to find that VLP deflation is very well represented (best fit of > 0.9) by a conduit with a centroid located near to the SW crater and extending from 750 to 500 m above sea level (that is from 0 to 250 m below the craters). The epicentral location differs by about 200 m from previous semblance analysis^2,18^, and it is in agreement with the radial particle motion of the VLP onset (Fig. 5) and moment tensor inversion based on the same data set^55^. The source that best fits the observed displacements coincides with the last *L* = 250 m of the conduit and is associated with a volume change *ΔV* of ~ 33 ± 10 m^3^ (Fig. 5). These deflation volumes are in good agreement with previous results inferred by seismic^2,56^ and ground tilt modeling^48^.

Assuming that this volumetric change is exerting a perturbation of the shallow magma conduit, the volume change is equivalent to a decompression Δ*P*:

3 $$\Delta P=\frac{5}{3}\mu \frac{\Delta V}{\pi {a}^{2}L}$$

of 8.5 ± 3.6 × 10^3^ Pa, where *μ* = 1.3 GPa is the rigidity of the host rocks^47^, *a* = 87 m is the equivalent radius of the shallow reservoir inferred by modeling the last four effusive eruptions^32^. Notably, this decompression is equivalent to a force of ~ 10^8^ N which is in the same order of magnitude as the single force calculated by moment tensor inversion^2^.

At the explosive onset, the momentum transferred by the explosive process to the magma column can be described as the downward reaction force per unit area $\Delta P=\rho {U}^{2}$ produced by the eruptive jet (gas-ash-and-lava fragments) with density *ρ* ejected at a velocity *U*. Assuming that the pressure drop, calculated by quasi-static VLP displacement, represents this reaction force *ΔP*, for a mean exit velocity *U* of gas and fragments of 40 m/s (Ref.^21^), the plume bulk density is ~ 5 kg/m^3^, which is in line with previous estimates of plume (gas-ash-and-lava fragments) density at Stromboli^57^. This explains the good fit (R = 0.76) between the VLP vertical displacement and the infrasonic over-pressure (Fig. 4) produced by the acoustic volumetric flux *Qi* (Eq. 1). Ground displacement during the dilatation A2 phase of the VLP is representing the magma depressurization of the last ~ 250 m feeding conduit (i.e., shallow reservoir) in response to the gas released during Strombolian eruptions.

## Discussion

Quasi-static displacement modelling of vertical ground motion^54,57^, using an unprecedented seismic network deployment in very near field conditions, indicates that the source of seismic VLP is located within the crater area and it has an extension of ~ 250 m below the craters. Families of VLPs, also when associated with different explosive craters, have in the long-term^26^ the same back-azimuth which does not allow to discriminate between different craters (Figure S1). We argue that particle motion of VLPs at Stromboli is indicating the centroid representing the base of a shallow crystal-rich magma plug elongated in the NE direction with equivalent radius of 87 m^30,32^, common to all the craters, which is excited by the explosive process (Figure S1).

**Figure 6:**
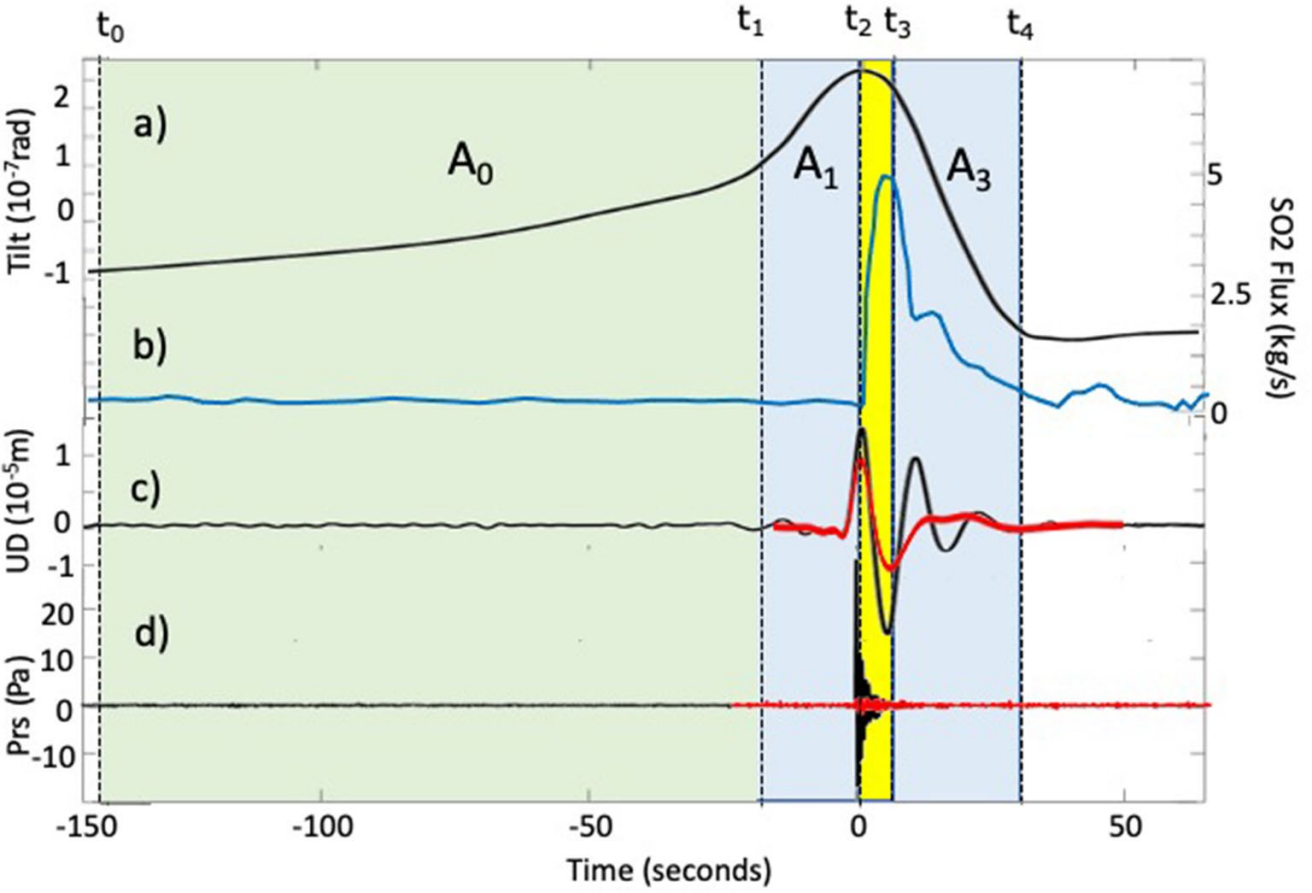
Strombolian explosion detected by ground tilt (**a**), SO_2_ flux (**b**), VLP seismic displacement (**c**) and infrasonic pressure (**d**). Ground tilt filtered between 0.001 and 0.02 Hz (**a**) reveals the almost 200 s-long charge and discharge gas process (*t*_0_–*t*_4_) controlling the explosive activity. Seismic VLP (**c**) begins during the final acceleration of the ground inflation (A1) ~ 15 s before the explosive onset (*t*_2_). During the explosion (yellow band), ground deflation correlates with the larger seismic VLP dilatation phase (A2 in Fig. 1) and with the acoustic signal (**d**). The pressure drop induced by the explosive jet (see also Fig. 7) triggers a ~ 20–30 s-long (*t*_3_-*t*_4_) deflation of the ground (**a**) and ﻿gas discharge phase (A3) as revealed by the UV camera^60^ (**b**) . Oscillations of the seismic VLP coda (A3 in **c**) reflects the reaction of the magma column to restore equilibrium. The larger the pressure drop is (VLP black line in **c**) the higher the amplitude of the acoustic pressure (black line in **d**) and the longer the oscillations are. VLP (**c**) and acoustic pressure (**d**) represent the stacking of family 1 (black line) and family 2 (red line) in Figure S1. Note that family 2 (red line) shows a smaller VLP amplitude (**c**), smaller acoustic pressure (**d**) and almost no oscillation in the coda (see supplementary material).

Thermal radiances and infrasonic waves show how the explosive onset is preceded by a small compressional VLP seismic phase (A1) lasting ~  < 5 s (Fig. 1), which is anticipating the explosion visible at the surface by several seconds (between 3 and 6 s). This time delay between the VLP (A1) and explosive onsets (*t1* in Fig. 6) suggests that seismic VLP originates before the explosion is occurring at the surface and it has been inferred to reflect the ascent, expansion, and burst of a gas slug within the last portion of the magma conduit^57^.

According to the “bottom-up” model^2^, gas rising from depth^44^ pushes up the magma within an inclined dyke (that resides at a moment-centroid constrained depth near 220—260 m below and ~ 160 m northwest of the vents)^2^, generating a volumetric expansion and the compressional onset (A1 in Fig. 1). This model, however, requires unrealistically fast fluid velocities of ~ 70 m/s^27^ to justify gas slug propagation from the VLP location to the surface (~ 250 m)^2,18^ in such a short time difference of ~ 3–5 s between the VLP and the explosion onset. The timing of the source dynamics observed at Stromboli is similar to that at other volcanoes when available video and/or acoustic sensors allow to associate the explosive process with VLP activity. At Kilauea^28^, Erebus^3,58^, Fuego^4^ and Aso^10^ volcanoes, VLP seismicity mainly develops after the onset of the explosion and the short time lag between VLP and explosion onsets is difficult to reconcile with the deep position of the seismic source using a classic fluid-dynamics model.

**Figure 7:**
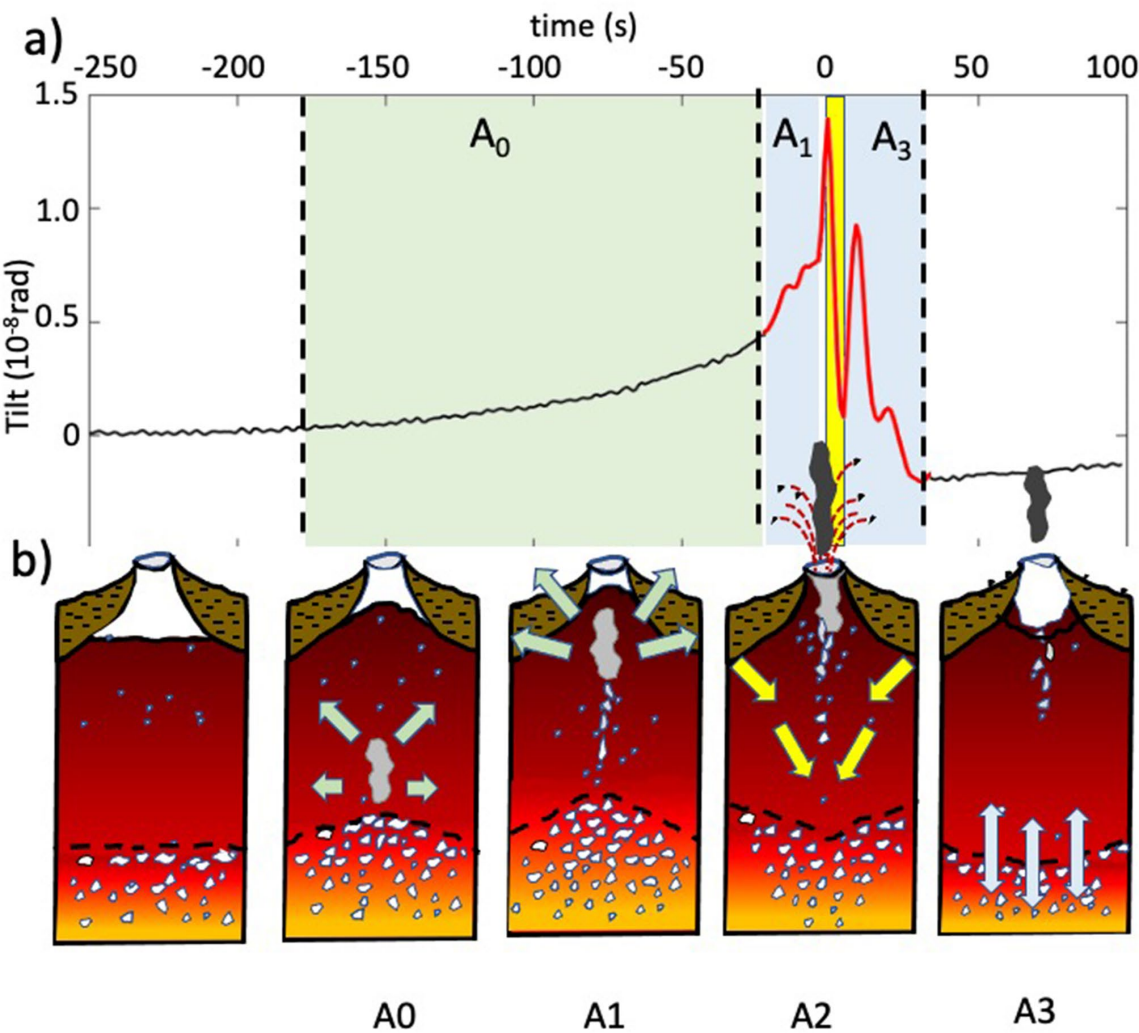
(**a**) Charge (A0 and A1) and discharge (A2 and A3) degassing cycle of the magmatic column revealed by the stacking of more than 1000 ground tilt events^47,54^. (**b**) Close-up of the shallow portion (last 300 m) of the conduit. Gas rich magma accumulating below a crystal-rich and denser magma mush^59^ (~ 150–200 s before the explosive onset) pushes up and deforms the shallow portion (< 250 m) of the magma column (A0 phase). The upward migration of a gas pocket through the mush slowly increases the pressure detected as an exponential trend of the ground deformation. Approaching the surface (~ 10–20 s before the explosive onset), gas migration and pressure drastically accelerate (A1 phase) triggering the explosion onset (at time 0 s). The mass flux ejected outside the vent is counterbalanced by a downward directed decompression phase (A2 phase yellow bar). At the explosive onset, the decompression associated with the gas discharge induces the drop of the magma level and the rebound of the magma column (A3 phase) during which magma column keeps degassing for 20–30 s after the end of the explosive process^63^. Seismic VLP (red segment in (**a**)) represents the final stage of this longer persisting charge/discharge ~ 200–250 s-long cycle (see also Fig. 2a) which releases the excess of gas accumulated at the base of a denser and degassed magma mush.

Ground tilt at Stromboli shows that the explosive phenomena is instead associated with a ~ 200 s (in average) long inflation-deflation deformation cycle. Ground starts to inflate ~ 150 s before the explosive onset (A0 in Figs. 6 and 7b) in response to the increase of the magmastatic head due to the uplift of a “cap”, driven by the expansion of the gas which accumulates below a crystal-rich and denser magma mush^59^, stagnant in the shallow portion (< 250 m) of the magma column^47,54^. While gas is expanding upwards in the magma mush (Fig. 7b), magmastatic pressure increases and ground inflation accelerates almost ~ 10 times in the last 10–20 s^47^ as the gas gets closer to the surface^54,60^ (A1 in Figs. 6 and 7b). Considering this dense crystal-rich mush is ~ 250 m thick, the duration of the inflation (~ 150 s) indicates that gas propagates at an average velocity of ~ 1.6 m/s^25^.

Seismic VLP sits on top of this inflation/deflation cycle and explosion occurs at the end of the ground inflation (Fig. 7a). The small compressional onset of the VLP represents only the final stage of the acceleration of the gas expansion just before the explosion. Laboratory experiments show, in fact, that when pressure of the gas overcomes the effective tensile strength of a granular suspension, gas propagates upwards inducing the inflation of the particle pack which accelerates while approaching the free surface^61,62^. Inflation/deflation tilt cycles associated with seismic VLP and explosive activity have also been observed at Fuego volcano and are explained as the pressurization/depressurization of the uppermost (~ 500 m) highly viscous plug of magma^4^. Similarly, in this silicic magma, seismic VLPs occur at the end of the inflation and are interpreted as the explosive depressurization of the conduit induced by the brittle failure of the viscous plug^4^.

Once the gas-fragments cloud is released in the atmosphere, magma level suddenly drops and the ground rapidly deflates^47,54^. Pressure inside the magma decreases, the conduit walls contract and the shallow crystal-mush moves downward to recover the equilibrium conditions (Fig. 7b). Ground deformation (Fig. 6a) shows that magma decompression persists (~ 30 s on average) longer than the duration of the explosions (~ 4 s on average) during which pressure oscillates (A3 in Figs. 1 and 6c) for 20–30 s before the magmastatic equilibrium is restored.

Oscillation of the VLP coda with ~ 20 s period and lasting several minutes have also been observed at Kilauea^13^, Erebus^3^ and Fuego^4^ volcanoes as associated with explosive phenomena and explained as induced by the magma recharge force^58^ and repressurization of the conduit^4^. The origin of the coda oscillations has been related to the dimension of the conduit or to the viscoelastic properties of the magma. At Stromboli, UV camera observations show that deflation of the ground matches with a ~ 30 s long SO_2_ gas emission following the explosion^63^ (Fig. 6b) and reflecting a long-lasting post-explosive decompression of the magma column. Coda oscillations are clear in the VLPs associated with large acoustic pressure (~ 40 Pa at 100 m) and are almost absent when VLP deflation is small and acoustic pressure low (~ 4 Pa at 100 m). This evidence (Fig. 6d and Fig. S1) suggests that when eruptive dynamics is linked to a small mass flux, the induced pressure drop is too small to trigger the oscillation of the shallow magma column. Our observations are therefore consistent with a “top-down” dynamic framework^28^, where VLP seismicity is reflecting only the final stage (< 20 s) of the charge/discharge degassing process of the magmatic column, and it represents a small perturbation in the long lasting (~ 200 s) pressurization cycles of the conduit (Fig. 7). This process repeats in time for every single explosion in a recurrent gas charge/discharge cyclic process.

The rate of VLP seismicity is in fact well correlated to the rate of explosions visible at the surface (Fig. 2). This correlation is lost only during effusive eruptions^18,30,32^, when the explosive activity at the summit craters stops. At the eruption onset the dip angle of VLP polarization moves downwards of several degrees (~ 10°), tracking the drainage of the shallow magmatic system^30–32^. Nevertheless, explosive degassing is still detected using SO_2_ imagery, at a rate that is well above the average (up to 60% of the total flux)^36^, suggesting that explosive activity is still on-going deep in the conduit. At the end of effusive eruptions, explosive activity progressively resumes at the summit crater, following the progressive refilling of the magma column within the conduit^30,31,64^, and the explosion-VLP correlation is then restored.

Our observations support the conclusion that VLP displacement wavefield is generated in a very shallow portion of the conduit at a rate proportional to the gas flux^36,61^ and it can be used to track the uppermost position of the magma column. Our result opens new pathways to the interpretation of seismic VLP source process at other open-vent basaltic volcanoes, and indicates that explosive activity can also occur when magma column is far from the surface and deep inside the conduit.

## Methods

### Experiment design and data acquisition

The geophysical data presented in this work are the result of experiments specifically designed to disclose the connection between VLP (0.05–0.2 Hz) seismicity and explosive activity. We used temporary stations located at the closest realizable distances to the craters and with the best azimuthal coverage possible considering safety constraints. These results are integrated with data from the permanent stations of the network operated by the Laboratory of Experimental Geophysics (LGS) of the University of Florence (http://lgs.geo.unifi.it) since 2003^65^. Seismic broad-band stations, thermal cameras, and acoustic stations were placed at distances ranging from < 100 m to 500 m from Stromboli’s active craters (Figure S2). All stations were synchronized using specific calibration tests and sampled at a variable rate according to the nature of the parameters but with the aim to guarantee a minimum of 0.01 s time accuracy and consistency.

We used a total of nine seismic stations consisting of Guralp CMG-40T broadband seismometers, with 30 s and 60 s eigenperiods and sensitivities of 800 V/m/s. All seismic stations were provided with differential acoustic pressure transducers with sensitivities of 25 mV/Pa, flat frequency responses between 0.01 and 100 Hz, and a full-scale range of 250 Pa. Together with the 5-element permanent infrasonic array, the acoustic wavefield was recorded with a network of 15 acoustic stations^43^. Ground deformation was measured using the 3 permanent tiltmeters placed in ~ 5 m depth boreholes sampled at 1 Hz with a nominal sensitivity of 1 nanoradian^47,54^.

Thermal images were recorded from the ROC (permanent) and PSF sites (Figure S2) with a *FLIR-A20* thermal camera fitted with a 34° × 25° optical lens (9.2 mm), and 0.1 °C thermal resolution, with focal plane array of 160 × 120 pixels, and sensitive in the 7.5–13 µm spectral range. Thermal images from PSF were collected at a frame rate of 50 Hz and were synchronized using a GPS clock with an accuracy of ~ 5 ms. The PSF thermal camera was located in direct line of sight with the NE and SW craters at a slant distance of 345 m and 327 m, respectively, and thus with field of views of 218 × 153 m and 206 × 145 m^35^*.*

### VLP localization using open conduit deformation model

The source parameters associated with VLP displacement field were estimated by integrating VLP ground velocity signals recorded at five temporary broadband seismic stations (yellow dots in Figure S2) together with the permanent stations (yellow square in Figure S2) of the LGS multiparametric network^54^.

Following an open conduit deformation model^54,57^, for an ideal Poissonian solid (Poisson modulus n = 0.25), the vertical displacement *u*_*z*_ is related to the volumetric change ΔV of a portion $L={z}_{2}-{z}_{1}$ of a cylindrical conduit by Eq. (2), where ${z}_{1}$ and ${z}_{2}$ are the top and the bottom depths, respectively. The Green’s function *G* in Eq. (2) is relative to the station *i* located at a distance *x* from the conduit and is defined as^53,60^:

4 $$G=\frac{-1}{2\left({z}_{2}-{z}_{1}\right)}\left[\frac{-{z}_{2}^{2}\left({z}_{2}-{z}_{1}\right)}{{R}_{2}^{3}}+\left(2\nu -1\right)\left(\frac{{z}_{2}}{{R}_{2}}-\frac{{z}_{1}}{{R}_{1}}\right)-2\nu {z}_{1}\left(\frac{1}{{R}_{2}}-\frac{1}{{R}_{1}}\right)-\left(2\nu -1\right)ln\left(\frac{{z}_{2}+{R}_{2}}{{z}_{1}+{R}_{1}}\right)\right]$$

where ${R}_{n}={\left({x}^{2}+{z}_{n}^{2}\right)}^\frac{1}{2}$ are the associated slant distances between the top (*n* = 1) and the bottom (*i* = 2) of the conduit and the station (Figure S3).

We applied a search algorithm for the best source conduit position within a 10 m-spaced grid of 900 × 450 m centered on the summit craters and extending on the Sciara del Fuoco, and assuming that *z*_*1*_ can vary, with step of 10 m, within 0 and 10,000 m below the surface, and *L* can vary between 10 and 1000 m with steps of 10 m. The best solution is thus estimated through a least square minimization among all the possible solutions between the ratios of the Green functions *G*_*n*_*/G*_*o*_ and the measured VLP displacement *u*_*i*_*/u*_*o*_ at the *i* station, where *G*_*o*_ and *u*_*o*_ are relative to the reference station. Once the best source and conduit positions as well as the conduit length *L* are found, the volumetric change Δ*V* is calculated by the slope of Eq. (2). Predicted VLP displacements differ by less than 10% respect to the measured VLP displacements, highlighting a strong linear correlation (R > 0.9) between source solution and observations (Fig. 5).

Our open conduit deformation model does not account for topographic effects on the measured VLP displacements. However, Finite Element Modeling (FEM) accounting for topography returned very comparable results, suggesting that while topography may have a large effect on displacement direction, it plays minor role (< 15%) in the associated amplitude^54^.

## Supplementary Information

Supplementary Information.

## Data Availability

All data generated or analysed during this study are included in this published article (and its Supplementary Materials) or available from the corresponding author upon reasonable request.
