# Supplementary material for: Magma pressure discharge induces very long period seismicity
Source: Sci Rep. 2021 Oct 8;11:20065. doi: 10.1038/s41598-021-99513-4 (PMC8501111; doi:10.1038/s41598-021-99513-4)
Supplement: Supplementary file 1 — Supplementary Information. [file 41598_2021_99513_MOESM1_ESM.pdf]

Supplementary Material 1

Seismic VLP waveforms

Two main waveforms (a and b) have been identified during the September 2016 experiment. We calculated the stacking from 620 and 522 VLP signals (grey lines) recorded at PZZ seismo-acoustic broadband station and generated by explosions from (a) SW and (b) NE craters, respectively. Despite the exploding vent are different, both clusters of VLP waveform show strong similarities, with an identical backazimuth (291°N) and dip pointing to the center of the crater area. The main difference between the two clusters is limited to the coda, which is more prolonged in signals associated with explosions (b) from NE better than (a) from SW vents. Notably, infrasonic signals produced by the NE crater explosions (d) are more energetic than those generated by SW crater (c) which are detected only at the stations S11 (shown in c and d) and S02 both located in the Fossa at <100 m from the vent. The two VLP clusters share the same backazimuth (e) and vertical dip (f) at all the stations indicating that seismic source is independent on the exploding vent and explosions nucleate in the same location.

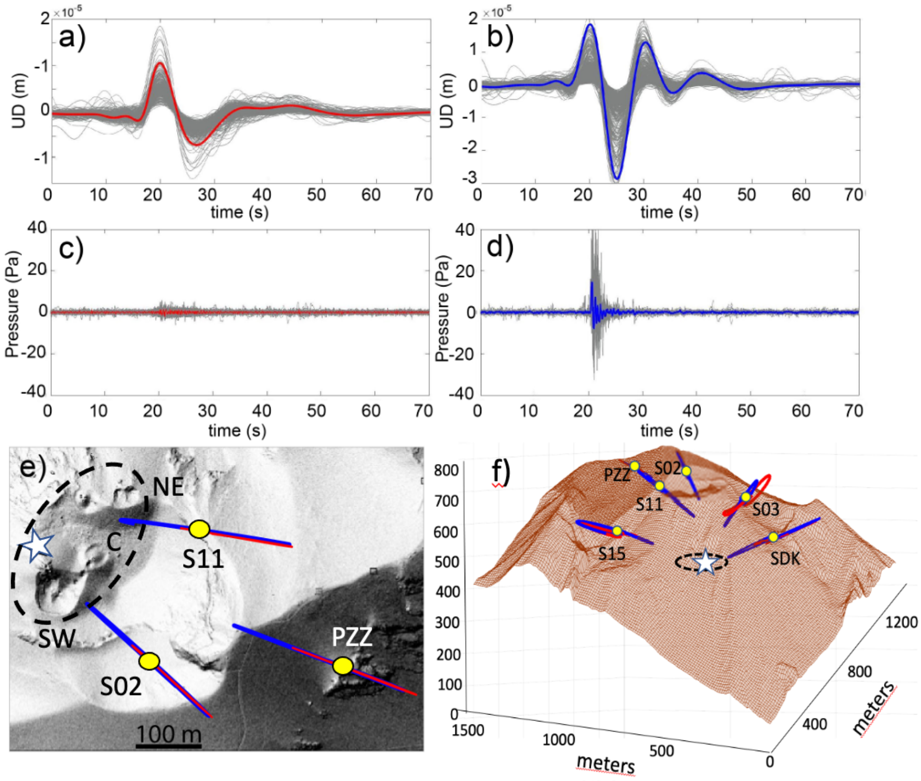

Figure S1. Stacking using a) 620 and b) 522 VLP signals (grey lines) recorded during the 24-30 Sept. 2016 experiment at the PZZ broadband station and generated by explosions from a) SW (red line) and b) NE (blue line) craters, respectively. Each VLP is associated with infrasonic signal (c, d) indicating the link with the explosive process. Particle motion calculated on the stacking waveform derived from the two clusters highlights the same e) backazimuth, and f) polarization dip. White star in e) and f) shows the position of the source location (Figure 5) calculated by grid search analysis using the open-conduit model (Supplementary Note 3). Dashed line locates the position (e) of the feeding conduit (see Figure S2) and the position of the base (f) of crystal-rich magma mush (Figure 7b).

Supplementary Material 2

Stromboli's network

Here we show the station positions whose data have been used in this work. Stations are subdivided between permanent and temporary ones, and among different types of measurement. Permanent stations located at the summit part of the LGS (http://lgs.geo.unifi.it/) multi-parametric surveillance network, installed since 2003[54,65], while the temporary stations have been deployed during specific field campaigns. In particular, high-frame rate thermal data of Figure 3 have been collected between 5 and 9 May 2008 from PSF[35], while VLP source location is mainly based on temporary stations (S02-S15) installed in September 2016 within the crater terrace. Long-term thermal and VLP correlations shown in Figure 2 have been observed from ROC permanent site.

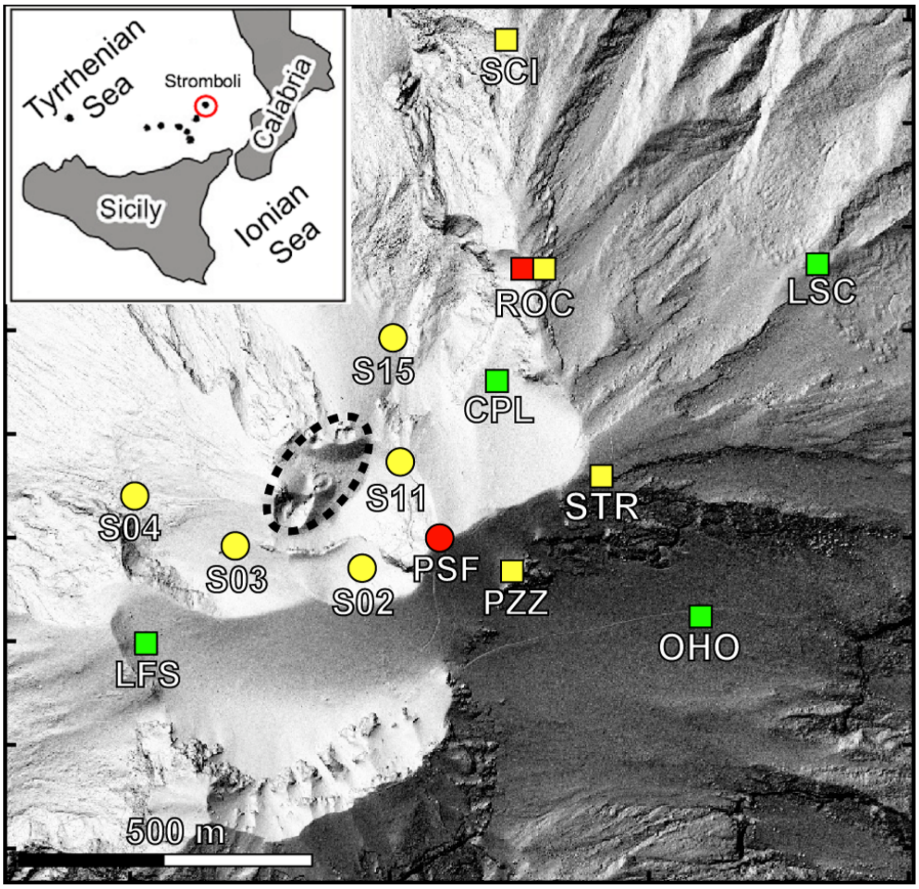

Figure S2. Map of Stromboli showing permanent (squares) and temporary (circles) station positions whose data have been used in this work, and subdivided among tiltmetric (green), seismo-acoustic (yellow) and thermal (red) stations. Map was created using Matlab ver.7.5.0.338 (R2007). Station names specifically mentioned in the main text are highlighted. Black dashed line marks the position of the summit craters which corresponds to the position of the Stromboli's shallow feeding conduit[30,32,54].

Supplementary Material 3

Source location using open conduit deformation model

The source parameters associated with VLP displacement field were estimated by integrating the VLP ground displacement signals recorded at five broadband seismic stations (yellow dots in Figure S2) installed within the Sciara del Fuoco scarp during our temporary experiment together with the permanent stations (yellow squares in Figure S2) of the LGS-UNIFI multiparametric network[54,65]. Given the several km-long wavelength of the seismic VLP waveform, the recorded seismic displacement is described using a quasi-static displacement model[3,50] as it is representative of the source time function.

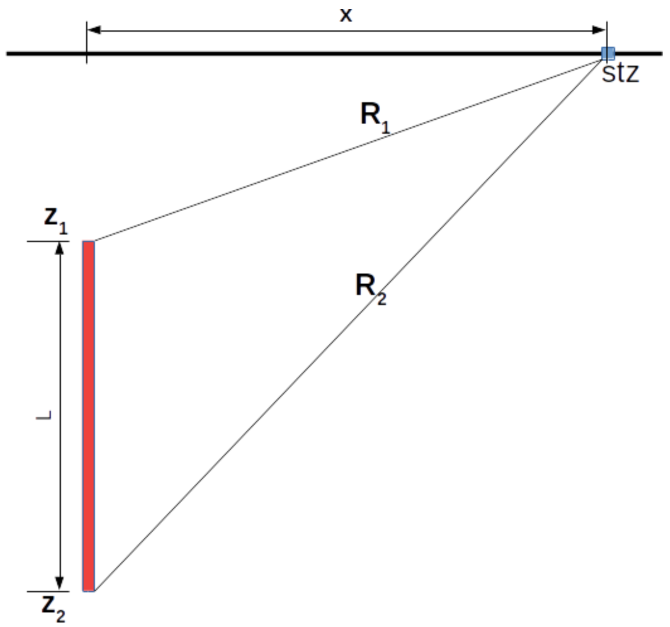

Figure S3. Schematic diagram showing the geometrical parameters defined for the open conduit inversion (redrawn from ref.[60])
